# Exosomal microRNA-22-3p alleviates cerebral ischemic injury by modulating KDM6B/BMP2/BMF axis

**DOI:** 10.1186/s13287-020-02091-x

**Published:** 2021-02-05

**Authors:** Yamei Zhang, Junying Liu, Mi Su, Xin Wang, Chenchen Xie

**Affiliations:** 1 Clinical Genetics Laboratory, Affiliated Hospital & Clinical Medical College of Chengdu University, No. 82 North Second Section of Second Ring Road, Jinniu District, Chengdu, 610081 Sichuan Province People’s Republic of China; 2 Department of Neurology, Affiliated Hospital of Chengdu University, Chengdu, 610081 People’s Republic of China

**Keywords:** Adipose-derived mesenchymal stem cells, Ischemia-reperfusion, Extracellular vesicles, microRNA-22-3p, KDM6B, BMP2, BMF

## Abstract

**Background:**

Cerebral ischemia-reperfusion (I/R) injury, the most common form of stroke, has high mortality and often brings persistent and serious brain dysfunction among survivors. Administration of adipose-derived mesenchymal stem cells (ASCs) has been suggested to alleviate the I/R brain injury, but the mechanism remains uncharacterized. Here, we aimed at investigating the mechanism of ASCs and their extracellular vesicles (EVs) in the repair of or protection from I/R injury.

**Methods:**

We established the middle cerebral artery occlusion (MCAO) model and oxygen-glucose deprivation/reperfusion (OGD/RP) neuron model. ASCs or ASC-derived EVs (ASC-EVs) were co-cultured with neurons. RT-qPCR and Western blot analyses determined microRNA (miRNA)-22-3p, BMP2, BMF, and KDM6B expression in neurons upon treatment with ASC-EVs. Bioinformatics analysis predicted the binding between miR-22-3p and KDM6B. Using gain- and loss-of-function methods, we tested the impact of these molecules on I/R injury in vivo and in vitro.

**Results:**

Treatment with ASCs and ASC-derived EVs significantly alleviated the I/R brain injury in vivo*,* elevated neuron viability in vitro, and decreased apoptosis. Interestingly, miR-22-3p was upregulated in ASC-EVs, and treatment with EV-miR-22-3p inhibitor led to increased apoptosis and decreased neuronal. Of note, miR-22-3p bound to and inhibited KDM6B, as demonstrated by dual-luciferase reporter gene assay and Western blot assay. Overexpression of KDM6B enhanced apoptosis of neurons in the OGD/RP model, and KDM6B bound to BMB2 and promoted its expression by binding to BMP2. Silencing of BMF reduced infarct volume and apoptosis in the stroke model.

**Conclusion:**

Results support a conclusion that ASC-EV-derived miR-22-3p could alleviate brain ischemic injury by inhibiting KDM6B-mediated effects on the BMP2/BMF axis. These findings compelling indicate a novel treatment strategy for cerebral ischemic injury.

## Background

Stroke, the second-most cause of death worldwide, encompasses hemorrhagic stroke, but the majority of cases are caused by arterial occlusion causing ischemic injury [1]. Stroke results in acute neuronal cell death and focal brain inflammation, which aggravates secondary brain injury by exacerbating blood-brain barrier damage, microvascular failure, brain edema, and oxidative stress [2]. Thanks to technological innovations, the clinical management of ischemic stroke has greatly advanced, notably through the use of intravenous thrombolysis and endovascular thrombectomy, which reduces disability [3]. However, identification of pathways and molecules that participate in cerebral ischemia could reveal novel approaches to improve the clinical outcome [4]. In this regard, animal models mimicking human stroke such as the middle cerebral artery occlusion (MCAO) model enable the study on the pathogenesis of cerebral ischemia [5].

Mesenchymal stem cell (MSC) transplantation has been demonstrated to improve functional and pathological recovery in cerebral ischemia [6]. In the condition of cerebral ischemia, the MSC transplantation engenders the migration of endogenous neuronal progenitor cells and alleviates the neuroinflammation associated with acquired brain injury [7, 8]. Of note, administration of adipose tissue-derived MSCs (ASCs) leads to functional recovery of cerebral lesions and reduces apoptosis, yet promotes neurogenesis and proliferation [9]. Besides, treatment with ASCs could suppress inflammatory factors, decrease neurological severity, and reduce the brain infarction volume [10]. However, the mechanism by which ASCs alleviate cerebral ischemic injury remains uncharacterized, thus calling for further studies.

Extracellular vesicles (EVs), which originate from the endosomal system, are involved in multiple physiological and pathological processes [11]. EVs from MSCs (MSC-EVs) are highlighted to protect the brain from hypoxia-ischemia by suppressing cerebral inflammation [12]. Furthermore, MSC-EVs attenuate postischemic immunosuppression in the peripheral blood after ischemia, thus improving neurological impairment [13]. The use of MSC-EV, as an alternative to MSCs, confers several advantages such as a higher safety profile, lower immunogenicity, and the ability of the cells to cross biological barriers [14]. Mounting evidence demonstrates that EVs from ASCs (ASC-EVs) regulate immune responses and delay the progression of diseases as diverse as experimental autoimmune encephalomyelitis and breast cancer [15–17]. ASC-EVs are also indicated to suppress neuronal apoptosis and activate autophagy, thereby ameliorating cerebral ischemia-reperfusion (I/R) injury in the rat MACO model [18]. microRNAs (miRNAs), which are key components of EVs, contribute to cell-to-cell communication both locally and systemically, directly affecting the process of osteogenic and adipogenic differentiation of MSCs [19, 20]. For example, miRNA-22-3p was highlighted to exhibit a protective effect against cerebral I/R injury, where its overexpression inhibited inflammatory cytokines and the pro-apoptosis gene Bax [21]. Besides, miRNA-22-3p enhances the intrinsic regenerative abilities of primary sensory neurons [22]. Nevertheless, little is known about the role of miR-22-3p in cerebral ischemic injury and its interaction with EVs or ASCs. The KDM6B histone demethylase is noted to play a critical role in osteogenic commitment of MSCs by removing H3K9me3 and H3K27me3 [23]. Indeed, KDM6B is an epigenetic regulator that mediates transcriptional activation during differentiation of hematopoietic stem and progenitor cells as well as immune responses [24]. In one study, loss of KDM6B activity leads to depletion of phenotypic and functional hematopoietic stem cells, indicating that KDM6B is a key to stem cell self-renewal in response to inflammatory and proliferative stress [25]. JMJD3 (KDM6B) knockdown has been reported to improve neurological deficits and reduced infarct volume following ischemic injury [26]. Given this background, in the present study, we aimed to explore the mechanism by which miR-22-3p and ASC-EVs in concert with KDM6B functioned in cerebral ischemic injury, using the MACO and oxygen and glucose deprivation (OGD) models. Interestingly, our results suggested that ASC-EVs protect brain injury through transferring miR-22-3p and that miR-22-3p inhibits histone demethylase KDM6B to exert protective activity.

## Methods

### Ethical statement

All animal experiments were performed with the approval of the Animal Ethics Committee of the Affiliated Hospital of Chengdu University. The experiments involving animals followed the recommendations in the *Guide for the Care and Use of Laboratory Animals* of the National Institutes of Health.

### Animal model

Adult Sprague Dawley (SD) rats (male, 10–12 weeks, 250–300 g) purchased from the Hunan SJA Laboratory Animal Co., Ltd. (Hunan, China) were subjected to temporary focal MCAO to induce focal ischemia [18]. In brief, rats were fixed on a stainless steel operating table under anesthesia with 30 mg/kg sodium pentobarbital (Sigma-Aldrich, St. Louis, MO, USA). The rat left internal carotid was exposed, and a nylon suture was inserted and advanced through the carotid bifurcation until the origin of the middle cerebral artery was blocked. After occlusion for 1 h, the suture was removed, followed by reperfusion. The sham-operated rats were subjected to the same surgery except without MCAO. Before the surgery, EVs (100 μg/kg/day) and siRNA (5 nmol/20 g/day) were injected via the lateral cerebral ventricle daily, and again for three consecutive 3 days. Three days after reperfusion, the rats were euthanized for the following experiments.

### Isolation of ASCs

After surgery, an adipose tissue sample was collected from normal SD rats and was treated as previously described [27]. In brief, the adipose tissue was minced and digested with 0.1% collagenase A (Roche Diagnostics, Mannheim, Germany) in PBS containing 1% bovine serum albumin (BSA; Roche Diagnostics). After Ficoll density centrifugation (Lymphoprep; Axis-Shield, Oslo, Norway), the cells were seeded (100,000 cells/cm^2^) into wells and cleaned of unattached cells by changing the medium. The ASCs were cultured in Dulbecco’s modified Eagle medium (DMEM) containing 10% fetal bovine serum (FBS; 30067334, Thermo Scientific, Waltham, MA, USA). Flow cytometry was performed to assess the expression of ASC surface marker. In brief, ASCs were first digested by trypsin with the addition of 10% goat serum (16210064, Thermo Scientific, Waltham, MA, USA) to prevent unspecific binding. Then, the ASCs were incubated with fluorescein isothiocyanate (FITC)-labeled primary monoclonal antibodies against CD73, CD44, CD90, CD34, HLA-DR, CD45, and CD166 (1:100, BioLegend, San Diego, CA, USA), with FITC-IgG as a control. Finally, the cells were washed with PBS, resuspended in goat serum, and analyzed by CyAn ADP Analyzer (Beckman Coulter, Brea, CA, USA).

### Phenotypic characterization of cultured ASCs

ASCs were placed in the DMEM medium with a specific solution [28] for 21–28 days with changing of the medium every 2 days. Then, the cells were fixed with 4% paraformaldehyde and washed with PBS, followed by examination for osteogenic, adipogenic, and chondrogenic differentiation potential by staining with Alizarin Red (2%), Alizarin Blue (1%), and Oil Red, respectively. Finally, the ASCs were observed and counted under an optical microscope (DM400, Leica, Wetzlar, German).

### Isolation of ASC-EVs

FBS for culturing ASCs was ultra-centrifuged at 1 × 10^6^*g* (Beckman Coulter Avanti J-30I, USA) for 16 h to obtain EV-free FBS, which was applied to the following experiment to avoid contamination from EVs. ASCs (approximately 3.2 × 10^7^ cells) at passages 2–3 were cultured in DMEM. When the confluence reached 70%, ASCs were continuously cultured in DMEM (with EV-free FBS) for another 24–48 h, and then the medium was collected and ultra-centrifuged to isolate ASC-EVs as previously described [29]. ASC-conditioned medium (CM) was centrifuged twice at 500×*g* for 10 min, twice at 2000×*g* for 15 min, and twice at 10,000×*g* for 30 min. The final supernatant was centrifuged again at 70,000×*g* for 1 h and washed with PBS to remove debris and large vesicles. The pellet was resuspended in 100 μL of PBS and stored at − 80 °C.

### Characterization of EVs

Analysis of the absolute size distribution of ASC-EVs was determined by dynamic light scattering (DLS) using Nanosizer™ instrument (Malvern Instruments, Malvern, UK). EVs were diluted in 1 mL of PBS and injected into the NanoSight NS300 instrument. Particles were automatically tracked and sized based on Brownian motion and the diffusion coefficient. Besides, the EVs were observed under a transmission electron microscope (Hitachi H7500 TEM, Hitachi, Tokyo, Japan) after staining with 1% uranyl acetate. Western blot analysis was further performed to determine the specific surface marker of EV expression, including rabbit antibodies against CD63 (ab216130; 1:2000, Abcam, UK), TSG101 (ab125011; 1:10,000, Abcam), and Calnexin (ab92573; 1:100,000, Abcam).

### Primary cortical neuron culture

As previously described [30], rat primary cortical neurons were obtained from newborn rats. In brief, cerebral tissues were minced and digested in trypsin for 30 min followed by the cell suspension being centrifuged at 3000×*g* for 10 min. The precipitate was resuspended in DMEM/F12 medium to adjust the cell concentration to 1 × 10^6^/mL. The cells were seeded into 96-well plates coated with 10 mg/L poly-l-lysine (Sigma, St. Louis, MO, USA) and incubated for 72 h. The cells then were incubated with arabinosylcytosine (Shanghai Yuanye Biotechnology Co., Ltd., Shanghai, China) in the medium for 24 h to suppress non-neuronal cell growth. Then, the cells were transferred to a normal medium, which was refreshed every 72 h. Immunofluorescence was performed to determine the expression of rabbit anti-MAP2 (A17409, 1:200, ABclonal, Boston, USA) and thus identify neurons. Oxygen-glucose deprivation/reperfusion (OGD/RP) was performed as described previously [21]. In brief, cortical neurons were treated with glucose-free Earl’s solution in 5% CO_2_ and 95% N_2_. At 72 h after treatment, cells were subjected to OGD/RP.

### EV internalization

Purified EVs were labeled with the Dil (Sigma-Aldrich). Neurons were seeded into 8-well chamber slides (Thermo Scientific™) at a density of 8 × 10^3^ cells/well and incubated with 5 μL of PKH67 for 24 h to allow internalization. After being washed with PBS twice, the neurons were fixed with 4% paraformaldehyde for 15 min, followed by staining with DAPI (0.5 mg/mL; Invitrogen, USA). Finally, the cells were photographed under a confocal microscope (Zeiss LSM 780; Zeiss, Jena, German).

### Infarct volume measurement

The brain was removed and sliced into six sequential coronal sections (± 5 mm, ± 3 mm, and ± 1 mm from the bregma). The sections were stained with 2% 2, 3, 5-triphenyltetrazolium chloride (TTC; Sigma) and fixed in 4% paraformaldehyde followed by photography with a digital camera (Kodak DC240, East-man Kodak Co., Ltd). Infarct volume was calculated according to the formula: lesion area of each section = (contralateral hemisphere area/ipsilateral hemisphere area) × ipsilateral lesion area. The volume of the lesion is estimated by multiplying the total areas of the lesion by the thickness of the slices [31].

### Neuronal nuclei (NeuN) immunofluorescence

The rat hippocampus section was pre-incubated with 0.3% Triton X-100 in PBS for 10 min and blocked with 0.1%Triton X-100 for 1 h. Primary rabbit anti-NeuN (1:500, ab177487, Abcam) and secondary goat anti-rabbit immunoglobulin G (IgG; 1:100, AS011, ABclonal, USA) were employed for immunofluorescence. The samples were placed in a DAPI matrix for nuclear staining and examined by inverted microscopy (Olympus IX71, Tokyo, China) in five randomly selected fields of the hippocampus CA1 region. Positive NeuN cells were counted by ImageJ software.

### Terminal deoxynucleotidyl transferase-mediated dUTP nick end-labeling (TUNEL) staining

TUNEL staining was performed to assess hippocampus apoptosis using the One-step TUNEL Apoptosis Assay kit (Green fluorescence) (Beyotime Institute of Biotechnology, Shanghai, China). In brief, 72 h after the establishment of the MCAO model, frozen sections of hippocampal tissue specimens were obtained. The sections were first fixed with 4% paraformaldehyde or Immunol Staining Fix Solution (P0098, Beyotime) for 30–60 min, followed by washing in PBS twice for 10 min. Sections were next incubated with PBS containing 0.5% Triton X-100 for 5 min and washed in PBS or HBSS twice. A total of 50 μL of TUNEL solution was added to the sections for 60-min incubation in the dark. After sealing the sections with anti-fluorescence quenching solution, the sections were observed under a fluorescence microscope.

### Cell transfection

Lentivirus vector pLVX-miR-22-3p mimic/inhibitor (Ambion, Carlsbad, CA); lentivirus packages overexpressed plasmids, OE-KDM6B, and OE-Bone morphogenetic protein 2 (BMP2) (GeneChem, Shanghai, China); and lentivirus-siRNA, si-KDM6B, and si-Bcl-2 modifying factor (BMF) (Guangzhou RiboBio Co., Ltd., Guangdong, China) were employed to treat the neurons. The neurons were then co-localized with ASC-EVs or CM or underwent OGD/RP. Moreover, in vivo siRNA (siBMF) experiments were performed with modified in vivo siRNA (Ribobio Guangzhou, China).

### Cell counting kit-8 assay (CCK-8) assay

The neurons were seeded into a 96-well plate at a density of 1 × 10^4^ cells/well. Optical density (OD) was measured at 450 nm using a microplate reader and CCK-8 kit (Dojindo Laboratories, Kumamoto, Japan). Cell viability was calculated as the following formula: viability (%) = experiment group (OD)/NC group (OD) × 100%.

### Dual-luciferase reporter gene assay

pGL3 enhancer vector (Genscript, Nanjing, China) was cloned into rat KDM6B-wild-type (WT)-3′untranslated region (3′UTR) or KDM6B-mutant (MUT)-3′UTR, where the binding site of miR-22-3p was included. HEK293T cells (from American Type Culture Center) were cultured in 24-well plates for 24 h and then co-transfected with luciferase reporter vector miR-22-3p mimic or NC mimic according to the instructions of Lipofectamine 3000 Reagent (Invitrogen, USA). After 48 h, the luciferase activity was detected by Dual-Luciferase Reporter Assay System.

### Chromatin immunoprecipitation (ChIP)

ChIP was conducted to quantify the enrichment of KDM6B and H3K27me3 in the BMP2 promotor region, using the detection kit (Millipore corp., Billerica, MA, USA). Specifically, the cells were crosslinked with 1% formaldehyde and then resuspended in SDS lysis buffer. After sonicating to disrupt the nucleus, protein A/G-beads was added to remove chromatin components. Then, anti-H3K27me3 antibody and anti-KDM6B antibody (ab38113, ABCAM) was added for incubation, with anti-rabbit IgG (ab171870, ABCAM), or anti-mouse IgG (ab81032, ABCAM) as NC. Finally, after decrosslinking and Proteinase K digestion, we used RT-qPCR to amplify and quantify the ChIP DNA, or IgG in the control samples. The primers for BMP2 were as follows: forward, 5′-CGTCTAGTATTTTGGCATAGCATAGACG-3′; reverse, 5′-ACTCAATTTCCAGCCTGCTGTTT-3′.

### Reverse transcription quantitative polymerase chain reaction (RT-qPCR)

Total RNA was extracted using Trizol Reagent (Invitrogen, Car, CA, USA) and reversely transcribed into cDNA with Revert Aid first-strand cDNA synthesis kit (Fermentas, Life Sciences, Canada). We utilized the SeraMir Exosome RNA Purification Kit (System Biosciences, Mountain View, USA) to isolate EV-miRNA. The synthesized cDNA was subjected to RT-qPCR based on the specifications of Fast Universal SYBR Green Master Mix (Roche, Indianapolis, USA) and ABI PRISM® 7900HT System (Takara Biotechnology, Japan), with the miRNA-specific forward primer (Sangon Biotech, Shanghai, China) and reverse primer from TaqMan microRNA assay kit (Table 1). In addition, we draw standard curves (2^−△△CT^) with GADPH as an internal reference. miR-22-3p in culture medium and EVs was normalized against the exogenous reference Cel-miR-39.

**Table 1:** Primer sequences for PCR

Genes	Sequence
miR-22-3p	F: 5′-GTGAAGCTGCCAGTTGAAG-3′
	R: 5′-GTGCAGGGTCCGAGGT-3′
Cel-miR-39-3p	F: 5′-UCACCGGGUGUAAAUCAGCUUG-3′
	R: 5′-A ACGCTTCACG A ATTTGCGT-3′
KDM6B	F: 5′-CAACTCCATCTGGCTGTTACTG-3′
	R: 5′-CCTTCTGCAACCAATTCCAG-3′
GAPDH	F: 5′-AGGTCGGTGTGAACGGATTTG-3′
	R: 5′-GGGGTCGTTGATGGCAACA-3′
BMP2	F: 5′-GGGACCCGCTGTCTTCTAGT-3′
	R: 5′-TCAACTCAAATTCGCTGAGGAC-3′
BMF	F: 5′-GGAGCGGGCGTATTTTGGAA-3′
	R: 5′-ACACTCGATTGGGAAGAAGGG-3′

*F* forward, *R* reverse

### Western blot analysis

Protein was separated using sodium dodecyl sulfate-polyacrylamide gel electrophoresis (SDS-PAGE) and transferred to polyvinylidene fluoride membranes (Immobilon P, Millipore, Billerica, USA), which were blocked in Tris-buffered saline containing milk (5%) and Tween-20 (0.1%) at room temperature for 1 h, followed by incubation with primary antibodies and horseradish peroxidase-labeled secondary antibody. Primary antibodies used in the experiment from ABclonal Biotechnology Co., Ltd. (Boston, USA) included rabbit antibodies against KDM6B (A17382, 1:2000), BMP2 (A5796, 1:2000), BMF (A5796, 1:2000), caspase 3 (A2156, 1:2000), cleaved caspase 3 (ab32042, 1:1500), Bax (A0207, 1:2000) and Bcl-2 (A0208, 1:2000), horseradish peroxidase-conjugated anti-rabbit IgG (AS014, 1:10,000), and anti-mouse IgG (AS003, 1:10,000). The bands were visualized using enhanced chemiluminescence reagents (Thermo Fisher Scientific, Waltham, USA) and images were taken by ChemiDoc XRS Plus luminescent image analyzer (Bio-Rad, CA, USA). Image-Pro Plus 6.0 software was applied to quantify band intensity, and the intensity of mouse anti-GAPDH (AC033, 1:50,000, ABclonal, USA) was applied to standardize target protein expression.

### Statistical analysis

The data were processed using SPSS 21.0 statistical software (SPSS Statistics, Chicago, IL, USA). Measurement data were presented as mean ± standard deviation. The data between two groups was analyzed by an independent *t* test. The data among multiple groups were analyzed by one-way analysis of variance (ANOVA) with Tukey’s post hoc test. *p < 0.05 was considered statistically significant.

## Results

### ASC-EVs are protective towards neurons under cerebral ischemic injury

**Fig. 1:**
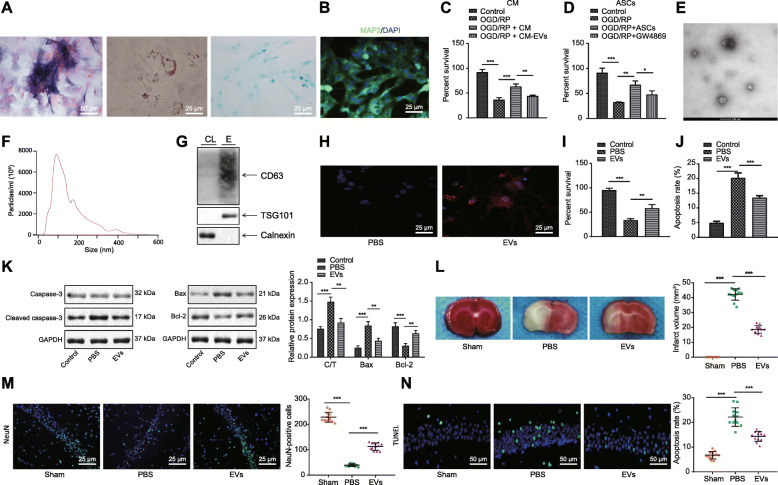
ASC-EVs protect neurons from ischemic injury. **a** Representative microscopic image of morphology of ASCs after osteogenesis, adipogenesis, and chondrogenesis. ALP for osteogenesis, Oil Red O staining for adipogenesis, and Alcian Blue staining for chondrogenesis (× 400). **b** Immunofluorescence of MAP2 expression in ASCs (× 400). **c** CCK-8 assay detection of cell viability of OGD/RP-induced neurons incubated in ASC-CM and EV-free CM, or controls. **d** CCK-8 assay detection of OGD/RP-induced neurons upon co-culture with ASCs and GW4869-treated ASCs, or controls. **e** Representative of EVs from ASCs under TEM (bar = 100 nm). **f** Quantification of size distribution of EVs under NTA. **g** Western blot analysis of specific EV-surface protein expression. **h** Representative images of neurons internalizing Dil-label EVs (red) and DAPI-labeled neuron nucleus (blue) (× 400). **i** CCK-8 assay detection of neuron viability upon treatment of ASC-EVs. **j** Flow cytometry detection of apoptosis upon ASC-EV treatment. **k** Western blot analysis of caspase 3, cleared caspase 3, Bax, and Bcl-2. C/T refers to the cleaved caspase 3/total caspase 3. **l** Representative macroscopic images of cerebral infraction volume with TCC staining. **m** NeuN immunofluorescence of hippocampus upon treatment of ASC-EVs, PBS, and sham operation (× 400). **n** Representative images of TUNEL staining of hippocampus apoptosis upon treatment of ASC-EVs, PBS, and sham operation (× 200). *p < 0.05, **p < 0.01, and ***p < 0.001. Measurement data were presented as mean ± standard deviation. The data among multiple groups were analyzed by ANOVA

ASCs have been reported to attenuate brain ischemic injury [32]. To further explore the involved mechanism, we first isolated ASCs and performed flow cytometry to identify classic MSC surface marker expression. We found highly expressed CD73 (99%), CD44 (96%), CD90 (97%), and CD166 (87%) as well as lowly expressed CD34 (5%), HLA-DR (0.5%), and CD45 (0.2%). Besides, the cells exhibited capacities of osteogenesis, adipogenesis, and chondrogenesis (Fig. 1a), confirming the effective isolation of ASCs. Meanwhile, rat primary cortical neurons were extracted successfully with purity over 95%, as identified by immunofluorescence (Fig. 1b). Next, the neurons incubated in CM containing ASCs or EV-free CM were subjected to OGD/RP, which decreased dramatically the neuron viability, which was rescued by the addition of CM (Fig. 1c). However, depletion of EVs in CM led to even lower viability. Next, neurons were indirectly co-cultured with ASCs or ASCs treated with GW4869, an inhibitor of exosome biogenesis/release, and subjected to GOD/RP treatment. This showed that the promoting effect of ASCs on neuron viability was inhibited by GW4869 (Fig. 1d). Collectively, ASCs could alleviate the neuronal response to the injury induced by OGD/RP, wherein EVs of ASCs played a key role.

To verify the protective effect of EVs, ASC-EVs were isolated and characterized. EVs were spherical and cup-shaped, with a diameter distribution around 100 nm (Fig. 1e, f). Western blot analysis further determined that the expression of CD63 and TSG101 increased in EVs (Fig. 1g), suggesting that EVs had been successfully isolated. Besides, with EVs labeled with Dil and neuron nucleus labeled with DAPI, the neurons were incubated with ASC-EVs for 24 h. Fluorescence microscopy showed that Dil-labeled EVs (red) were clustered around the neuronal nucleus (blue), reflecting the process of ASC-EV internalization (Fig. 1h). Apart from these findings, ASC-EVs remarkably increased viability and decreased the apoptosis induced by OGD/RP (Fig. 1i, j). It is well known that caspase 3 is a prominent mediator of apoptosis [33], while Bax and Bcl-2 are apoptosis-related genes [34]. Thus, we analyzed the effects through determination of the expression of caspase 3, Bcl-2, and Bax. Results showed that cleaved caspase 3/caspase 3 and Bax expression were decreased and Bcl-2 expression was increased (Fig. 1k). To investigate the in vivo effect of EVs, we established the MACO rat model and treated the rats with PBS or ASC-EVs for 3 days. The infarct volume and apoptosis were reduced and NeuN-positive cells were increased upon ASC-EV injection (Fig. 1l–n), thus indicating that ASC-EVs could alleviate the injury caused by OGD/RP.

### miR-22-3p derived from ASC-EVs could transfer to neurons

**Fig. 2:**
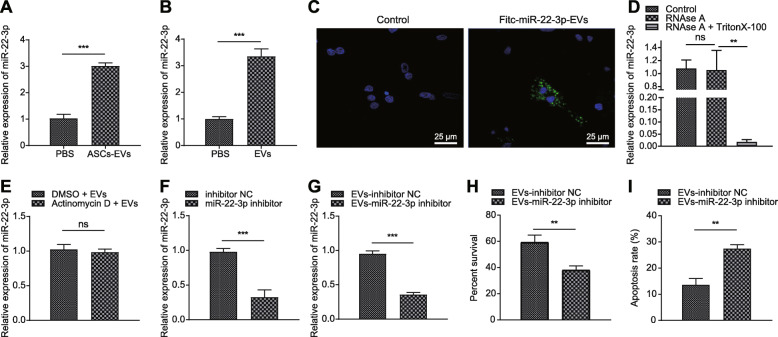
ASC-EVs alleviate cerebral ischemic injury through miR-22-3p. **a** RT-qPCR analysis of miR-22-3p expression in neurons. **b** RT-qPCR analysis of miR-22-3p expression in cerebral tissues. **c** Representative microscopic images of FITC-miR-22-3p mimics in neurons (× 400). **d** RT-qPCR analysis of miR-22-3p expression in neurons upon addition of RNase A in CM. **e** RT-qPCR analysis of miR-22-3p expression in neurons upon treatment of actinomycin D and ASC-EVs. **f** RT-qPCR analysis of miR-22-3p expression in ASCs upon miR-22-3p inhibitor. **g** RT-qPCR analysis of miR-22-3p expression in neurons incubated with the EVs from ASCs transfected with miR-22-3p inhibitor. **h** CCK-8 assay detection of neuron viability upon treatment of miR-22-3p inhibitor. **i** Flow cytometry detection of apoptosis upon treatment of miR-22-3p inhibitor. *p < 0.05, **p < 0.01, and ***p < 0.001. Measurement data were presented as mean ± standard deviation. The data among multiple groups were analyzed by ANOVA

Previous studies revealed that overexpressing miR-22-3p could alleviate the injury to neurons caused by I/R injury [21]. To test the prediction that ASC-EVs might exert a protective activity by transferring miR-22-3p, we performed a series of assays. First, incubation with ASC-EVs induced an increase of miR-22-3p expression in neurons and cerebral tissues (Fig. 2a, b). Besides, ASCs were transfected with FITC-labeled miR-22-3p mimic and their EVs were then isolated and used to incubate with neurons. Strong green fluorescence appeared in neurons incubated with ASC-EVs (Fig. 2c). Interestingly, the addition of RNase A to CM of ASCs hardly altered miR-22-3p expression, but miR-22-3p could not be detected upon treatment with RNase A + Triton X-100 (Fig. 2d). The fact that actinomycin D did not alter miR-22-3p expression excluded the possibility of endogenous induction (Fig. 2e), indicating that EVs or EV-derived miR-22-3p was internalized by neurons. Moreover, in the presence of the miR-22-3p inhibitor, apoptosis of neurons was increased, but their viability was decreased (Fig. 2f–i). Therefore, we conclude that ASC-EV-derived miR-22-3p (ASC-EV-miR-22-3p) could alleviate the response of neurons to ischemic injury.

### KDM6B is the target of miR-22-3p in neurons

**Fig. 3:**
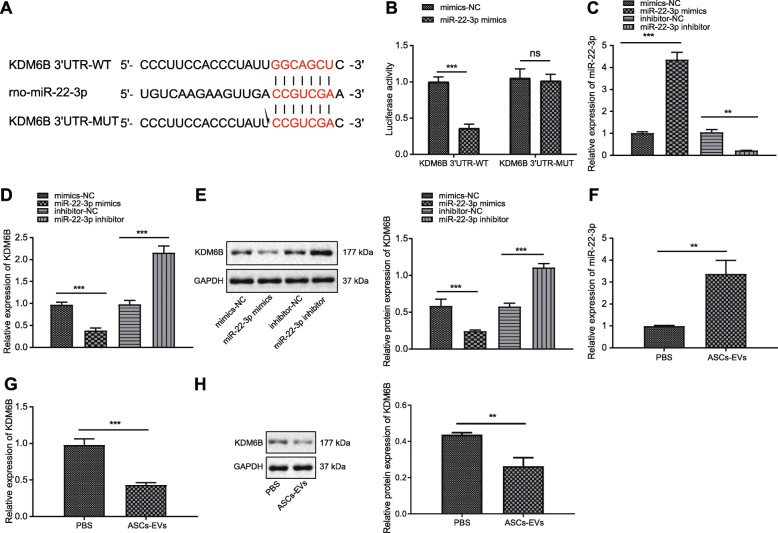
KDM6B is a target gene of miR-22-3p derived from ASC-EVs. **a** Specific binding site of KDM6B and miR-22-3p predicted by an online website. **b** Dual-luciferase reporter gene assay of KDM6B and miR-22-3p. **c** RT-qPCR analysis of miR-22-3p expression in neurons upon transfection of miR-22-3p mimic or inhibitor. **d** RT-qPCR analysis of KDM6B expression in neurons upon transfection of miR-22-3p mimic or inhibitor. **e** Western blot analysis of KDM6B expression in neurons upon transfection of miR-22-3p mimic or inhibitor. **f** RT-qPCR analysis of miR-22-3p expression in neurons upon treatment of ASC-EVs. **g** RT-qPCR of KDM6B expression in neurons upon treatment of ASC-EVs. **h** Western blot analysis of KDM6B expression in neurons upon treatment of ASC-EVs. *p < 0.05, **p < 0.01, and ***p < 0.001. Measurement data were presented as mean ± standard deviation. The data among multiple groups were analyzed by ANOVA

To further explore the downstream regulatory system of ASC-EV-miR-22-3p in cerebral ischemic injury, we searched the TargetScan database to predict downstream genes of miR-22-3p, which revealed a binding site of miR-22-3p at KDM6B 3′UTR 127-133 (Fig. 3a). Indeed, KDM6B has been shown to be highly expressed during cerebral ischemic injury [26]. Therefore, it is reasonable to suppose that miR-22-3p might attenuate cerebral ischemic injury by inhibiting the expression of KDM6B. To confirm the prediction from TargetScan, we performed the dual-luciferase reporter gene assay, which showed that co-transfection of KDM6B 3′UTR-WT and miR-22-3p mimic led to decreased luciferase activity. Nevertheless, no such difference appeared in KDM6B 3′UTR-MUT. These alterations of luciferase activity indicated that miR-22-3p mimic specifically targeted at the 3′UTR region of KDM6B (Fig. 3b). Besides, functional experiment indicated that transfection of miR-22-3p mimic led to a decrease in the mRNA and protein expression of KDM6B and that miR-22-3p inhibitor evoked the opposite effect (Fig. 3c–e). Moreover, when neurons were incubated with ASC-EVs, miR-22-3p expression was increased and KDM6B expression decreased (Fig. 3f–h). Collectively, ASC-EV-miR-22-3p could target and inhibit KDM6B expression in neurons.

### KDM6B promotes cerebral ischemic injury

**Fig. 4:**
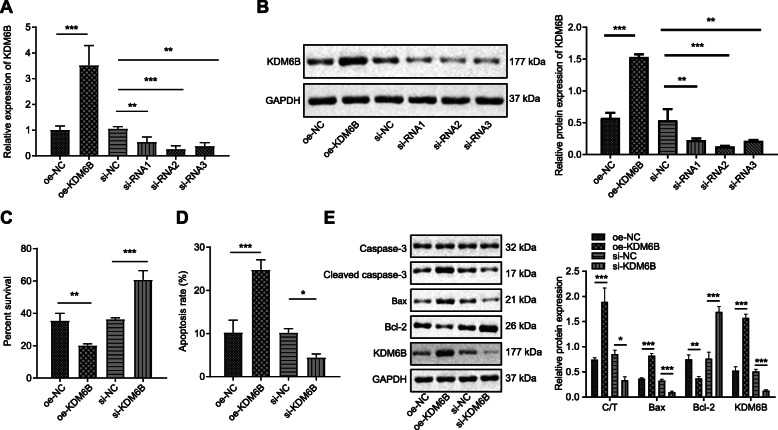
High KDM6B expression exacerbates cerebral ischemic injury. **a** RT-qPCR analysis of KDM6B expression in neurons upon treatment of OE-KDM6B or si-KDM6B. **b** Western blot analysis determining transfection effect of OE-KDM6B, siRNA1, siRNA2, siRNA3, and their corresponding controls. **c** CCK-8 assay detection of viability of neurons after treatment with OE-KDM6B or si-KDM6B, or controls. **d** Flow cytometry detection of apoptosis after treatment of OE-KDM6B and si-KDM6B, or controls. **e** Western blot analysis of cleaved caspase 3, caspase 3, Bax, and Bcl-2 protein expression after treatment of OE-KDM6B or si-KDM6B, or controls, and the corresponding quantification. *p < 0.05, **p < 0.01, and ***p < 0.001. Measurement data were presented as mean ± standard deviation. The data among multiple groups were analyzed by ANOVA

To investigate the impact of KDM6B on cerebral ischemic injury, three siRNAs against KDM6B and the plasmids overexpressing KDM6B were transfected into neurons. Using RT-qPCR and Western blot analysis, we confirmed the efficiency of oe-KDM6B and si-KDM6B as well as siRNAs, with siRNA2 exhibiting the most significant inhibitory effect (Fig. 4a, b). In the presence of OE-KDM6B, apoptosis was increased and viability was decreased in OGD/RP-induced neurons, as shown by CCK-8 assay and flow cytometry (Fig. 4c, d). Moreover, cleaved caspase 3/caspase 3 and Bax expression were elevated, whereas Bcl-2 declined (Fig. 4e). Collectively, the aforementioned evidence implies that KDM6B facilitated neuron apoptosis upon OGD/RP.

### ASC-EVs protect neurons by inhibiting KDM6B expression

**Fig. 5:**
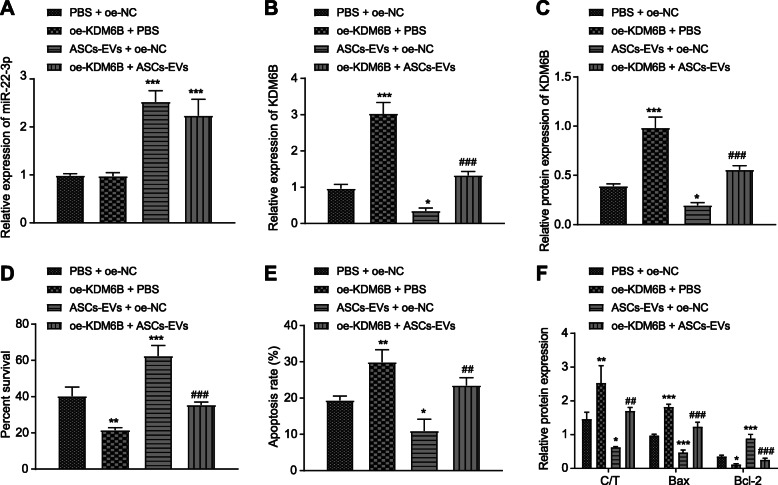
Overexpressed KDM6B limits the protective effect of ASC-EVs on neurons. **a** RT-qPCR analysis of miR-22-3p expression in neurons upon treatment of ASC-EVs, OE-KDM6B, or PBS. **b** RT-qPCR analysis of KDM6B expression in neurons upon treatment of ASC-EVs, OE-KDM6B, or PBS. **c** Western blot analysis of KDM6B expression in neurons upon treatment of ASC-EVs, OE-KDM6B, or PBS. **d** CCK-8 assay detection of cell viability of neurons upon treatment of ASC-EVs, OE-KDM6B, OE-NC, or PBS. **e** Flow cytometry detection of apoptosis of neurons upon treatment of ASC-EVs, OE-KDM6B, OE-NC, or PBS. **f** Western blot analysis of cleaved caspase 3, caspase 3, Bcl-2, and Bax expression in neurons upon treatment of ASC-EVs, OE-KDM6B, OE-NC, or PBS. *p < 0.05, **p < 0.01, and ***p < 0.001. Measurement data were presented as mean ± standard deviation. The data among multiple groups were analyzed by ANOVA

To test the direct relation between ASC-EVs and KDM6B, neurons were transfected with OE-KDM6B and/or incubated with ASC-EVs. Results showed that treatment of ASC-EVs relative to PBS control remarkably increased miR-22-3p expression and decreased KDM6B expression in neurons, while OE-KDM6B + PBS relative to OE-NC + PBS increased KDM6B expression. Compared to the KDM6B level in ASC-EVs + OE-NC-treated neurons, the concentration was higher in ASC-EVs + OE-KDM6B-treated neurons (Fig. 5a–c). Besides, according to CCK-8 assay and flow cytometry, OE-KDM6B treatment remarkably inhibited viability and enhanced apoptosis, while ASC-EV treatment had the opposite effect (Fig. 5d, e). OE-KDM5B + ASC-EVs treatment further inhibited apoptosis and increased viability. ASC-EV treatment clearly inhibited cleaved caspase 3, caspase 3, and Bax expression but increased Bcl-2 expression, whereas treatment with OE-KDM6B had opposite effects (Fig. 5f). Taken together, we find that overexpression of KDM6B could reduce the protective effect of ASC-EVs on neurons.

### KDM6B promotes BMF expression via regulating BMP2

**Fig. 6:**
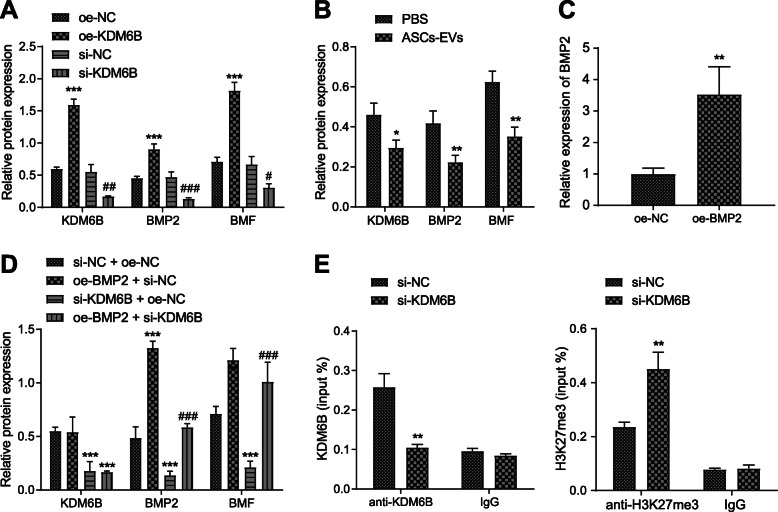
KDM6B promotes the expression of BMP2/BMF axis. **a** Western blot analysis of the expression of KDM6B, BMP2, and BMF in neurons upon treatment of OE-KDM6B or si-KDM6B. *p < 0.05, **p < 0.01, and ***p < 0.001. ^#^*p* < 0.05 vs. si-NC treatment. ^##^*p* < 0.01 and ^###^*p* < 0.001. **b** Western blot analysis of the expression of KDM6B, BMP2, and BMF in neurons upon co-culture with ASC-EVs or PBS. *p < 0.05 vs. PBS, **p < 0.01, and ***p < 0.001. **c** RT-qPCR analysis determining the transfection of OE-BMP2 in neurons. **d** Western blot analysis of KDM6B, BMP2, and BMF expression in neurons upon treatment of OE-BMP2, OE-NC, si-KDM6B, or si-NC. *p < 0.05, **p < 0.01, and ***p < 0.001. ^#^*p* < 0.05 vs. si-KDM6B + OE-NC treatment. ^##^*p* < 0.01 and ^###^*p* < 0.001. **e** ChIP assay detection of the binding between KDM6B and BMP2 enhancer region or H3K27me3 modification upon treatment of si-KDM6B. *p < 0.05 vs. si-NC treatment, **p < 0.01, and ***p < 0.001. Measurement data were presented as mean ± standard deviation. The data between two groups were analyzed by an independent *t* test. The data among multiple groups were analyzed by ANOVA

Previous studies have noted that KDM6B could bind to BMP2 enhancer and promote BMP2 expression by depleting H3K27me3 [35]. Besides, BMP2 is implicated to be highly expressed in cerebral ischemic injury and could enhance BMF expression [36, 37]. Therefore, we next examined the possibility that KDM6B promotes ischemic injury by BMP2/BMF regulation. Upon treatment of OE-KDM6B, BMP2 and BMF expression was increased, and conversely, si-KDM6B decreased the expression of BMP2 and BMP (Fig. 6a). Afterwards, we found that co-culturing neurons with ASC-EVs decreased the expression of KDM6B, BMP2, and BMP (Fig. 6b). Further, to check the interaction among KDM6B, BMP2, and BMP, we applied plasmids of OE-BMP2 and si-KDM6B, with confirmation by RT-qPCR of their inhibitory or mimicking activity in neurons (Fig. 6c). After neurons were simultaneously transfected with si-KDM6B and BMP2, Western blot analysis was conducted to detect the expression of KDM6B, BMP2, and BMF (Fig. 6d). Results showed that, compared to control treatment, OE-BMP2 + si-NC treatment elevated the expression of BMP2 and BMF, while si-KDM6B + si-NC treatment decreased the expression of KDM6B, BMP2, and BMF. Relative to si-KDM6B + si-NC treatment, the combination of si-KDM6B and OE-BMP2 increased the BMP2 and BMF levels, reversing the inhibitory effect of si-KDM6B on BMF. These data suggest that KDM6B increased BMF expression through mediating effects on BMP2 expression. To identify further this potential regulatory mechanism, we silenced KDM6B expression in neurons and carried out ChIP experiments to detect the binding between KDM6B and BMP2 promoter, also with H3K27me3 modification in the enhancer region (Fig. 6e). Results showed that si-KDM6B remarkably inhibited the binding between KDM6B and the BMP2 promoter region but increased H3K27me3 modification in the enhancer region. Taken together, KDM6B depleted the H3K27me3 modification of BMP2 and promoted its expression, thereby enhancing BMF expression through binding to BMP2 enhancer region.

### ASC-EV-miR-22-3p alleviates cerebral ischemic injury through KDM6B-mediated BMP2/BMF axis

**Fig. 7:**
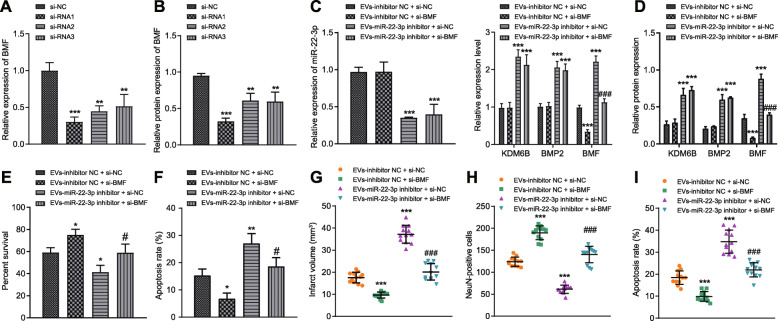
ASC-EV-miR-22-3p suppresses KDM6B-mediated BMP2/BMF axis and alleviates ischemic injury. **a** RT-qPCR analysis determining the efficiency of si-BMF (siRNA1, siRNA2, and siRNA3). **b** Western blot analysis determining the efficiency of si-BMF (siRNA1, siRNA2, and siRNA3). *p < 0.05 vs. si-NC, **p < 0.01, and ***p < 0.001. **c** RT-qPCR analysis of miR-22-3p, KDM6B, BMP2, and BMF mRNA expression in neurons upon treatment of si-BMF and EVs. ***p < 0.001 vs. EV-inhibitor NC + si-NC and ^###^*p* < 0.001 vs. EV-miR-22-3p inhibitor + si-NC. **d** Western blot analysis of miR-22-3p, KDM6B, BMP2, and BMF expression in neurons upon treatment of si-BMF and EVs. **e** CCK-8 assay detection of viability upon treatment of EV-inhibitor NC, siBMF, EV-miR-22-3p inhibitor, and si-NC. **f** Flow cytometry detection of apoptosis in neurons upon treatment of EV-inhibitor NC, si-BMF, EV-miR-22-3p inhibitor, and si-NC. **g** Cerebral infraction volume with TCC staining. **h** NeuN immunofluorescence of positive NeuN cells upon treatment of ASC-EVs, PBS, and sham operation. **i** TUNEL staining of neurons apoptosis upon treatment of ASC-EVs, PBS, and sham operation. From **f** to **i**, *vs. EV-miR-22-3p inhibitor + NC: *p < 0.05, **p < 0.01, and ***p < 0.001; ^#^vs. EV-miR-22-3p inhibitor + si-NC: ^#^*p* < 0.05, ^##^*p* < 0.01, and ^###^*p* < 0.001. Measurement data were presented as mean ± standard deviation. The data between two groups were analyzed by independent *t* test. The data among multiple groups were analyzed by ANOVA

To explore the mechanism whereby ASC-EV-miR-22-3p alleviates cerebral ischemic injury through BMF, we knock downed BMF expression in neurons through transfection with si-BMF. Since si-BMF1 exhibited the greatest inhibitory effect, this approach was selected for the following assays (Fig. 7a, b). Compared with EV-inhibitor NC + si-NC, si-BMF + EV-inhibitor NC treatment reduced BMF expression, yet EV-miR-22-3p inhibitor + si-NC treatment promoted KDM6B, BMP2, and BMF expression (Fig. 7c, d). EV-miR-22-3p inhibitor and si-BMF treatment led to a decreased in BMF. Moreover, based on CCK-8 assay and flow cytometry, EV-inhibitor NC + si-BMF treatment enhanced neuronal viability and suppressed apoptosis (Fig. 7e). EV-miR-22-3p inhibitor treatment resulted in lower cell viability and increased apoptosis. However, the addition of si-BMF rescued the effect caused by EV-miR-22p inhibitor (Fig. 7f).

Animal experiments were also performed to test the in vivo effects of perturbing this mechanistic axis. After MACO, rats were injected with si-BMF or EVs, which showed that EV-miR-22-3p inhibitor led to increased infarcted volume, whereas the addition of si-BMF reduced the volume of infarct (Fig. 7g). As such, EV-miR-22-3p inhibitor enhanced apoptosis and reduced the number of NeuN^+^ cells, which was reversed by the treatment of si-BMF (Fig. 7h, i). Therefore, we conclude that silencing BMF could alleviate ischemic injury. Taken altogether, ASC-EV-miR-22-3p alleviates cerebral ischemic injury by inhibiting the BMP2/BMF axis.

## Discussion

Upon the onset of cerebral ischemic injury, oxygen and glucose are transiently depleted, inducing a cascade of deleterious cellular events including accumulation of excitatory glutamate in the extracellular space [38]. Normally, some limited spontaneous functional recovery takes place upon I/R injury [36]. Considering the limited treatment options for cerebral ischemic injury, new effective treatment strategies to protect the brain from I/R injury are urgently required [38]. Although recent years have witnessed the failure in the development of thousands neuroprotective drugs, neuroprotection based on novel pathways could still be attainable in stroke patients. In the present study, we demonstrated that ASCs alleviated the brain injury and reduced neuron apoptosis through EV-miR-22-3p.

MSCs can improve neuronal survival by promoting the anti-apoptotic signaling cascade, whereby paracrine factors secreted by MSCs protect neurons from apoptotic cell death in the OGD model of cerebral ischemia [39]. Besides, MSCs decreased OGD-induced apoptosis and inflammation after ischemic injury [40]. Systemic administration of MSC-EVs improved brain function by reducing seizures and preserving baroreceptor reflex sensitivity in a stroke model [12]. Even when administered after transient global cerebral ischemia, ASCs exhibited a prominent protective effect on neuron death [41]. Interestingly, auto-ASCs were more effective than allo-ASCs in reducing the infarct volume of MCAO rats [32]. Besides, ASC-CM could rescue normal axonal morphology, electrophysiological features, and cell viability upon ischemic injury [42]. In the present study, the protective effect of ASCs on brain injury was confirmed and, moreover, ASC-CM with depletion of EVs was not effective as ASC-CM. This supported the potential role of EVs of ASCs in ischemic injury, which was further indicated by our finding that injection of EVs into MCAO rats significantly reduced the infarct volume. Likewise, MSC-EVs have been previously indicated to recover the impaired function and structural injury of brain upon hypoxia-ischemia [12]. Mechanistically, EVs induce long-term neuroprotection, neurological recovery, and favorable immune responses following ischemia [13].

Furthermore, our work unraveled that miR-22-3p from ASC-EVs attenuated apoptosis and brain injury by inhibiting KDM6B expression. Certain miRNAs such as miR-124 have been highlighted for their role in modulating signaling cascades to regulate stroke pathology, while these miRs promote or inhibit vascular endothelial cell biology and angiogenesis, which directly affects the progression of cerebral ischemia [43]. miRNAs from ASC-EVs have been indicated to attenuate ischemic brain injury. For example, ASCs were shown to suppress inflammation and protect against brain injury by suppressing miR-21-3p, which directly inhibits protein expression [44]. In addition, miR-22-3p overexpression facilitated M2 polarization of macrophages and inhibited inflammation, thereby attenuating I/R injury [45]. Moreover, caspase-3 activity and Bax were inhibited by miR-22, and the expression of Bcl-2 in neurons was increased, such that miR-22 could protect against cerebral I/R injury [21]. Similarly, the current study also found that overexpressing miR-22-3p reduced the brain infarct volume, suppressed apoptosis, and enhanced viability of neurons.

Cerebral I/R injury may cause neurological impairment in conjunction with caspase-3 and Bax activation as well as Bcl-2 downregulation [46]. KDM6B (JMJD3) interacts with and recruits co-activators and transcription factors in the promoter region of the target genes to activate transcription [47]. JMJD3 is a critical promoter of neuronal apoptosis by regulating Bax as well as caspase-3, where silencing JMJD3 could improve neurological deficit and reduce the ischemic injury [26]. Consistent with these findings, our present results indicate that KDM6B worsened cerebral ischemic injury by activating caspase-3 as well as Bax, while deactivating Bcl-2. Importantly, JMJD3 catalyzes the transition of H3K27me3 to H3K27me, thereby maintaining homeostasis by osteoblast differentiation, maturation, and apoptosis [48]. Mechanistically, KDM6B epigenetically activates neuronal genes by removing the repressive chromatin marker histone H3 lysine 27 trimethylation [49]. The present study unraveled that KDM6B promoted BMP2 expression by binding to the promoter region of BMP2 and depleting H3K27me3 modification. Similar to the present results, silencing KDM6B diminished the binding pattern of KDM6B in the BMP2 promoter regions in MSCs [27]. Under conditions of hypoxia, an increased level of H3K27me3 on the promoter region of BMP2, in combination with downregulation of KDM6B activity, suppressed osteogenic phenotypes of human periosteum-derived cells [50]. Another major finding of our study was that KDM6B promoted ischemic injury by enhancing BMF expression through BMP2. BMP2 is a neurotrophic factor, which induces the growth of midbrain dopaminergic neurons in vitro and in vivo, exerting neurotrophic effects [51]. Bone marrow MSCs increase BMP2/4 expression in ischemic astrocytes, enhancing subventricular progenitor cell gliogenesis by activating relevant signaling pathways and thus improving functional recovery after stroke [52]. Previous studies depicted that BMP2 expression was increased in the ischemic brain for as long as 4 weeks [36]. BMP-2 induces differentiation of ASCs into chondrocyte-like cells when promoting osteogenic gene expression levels and alkaline phosphatase activity in ASCs [53, 54]. On the other hand, BMF provokes damage to the cytoskeleton and facilitates cytochrome formation [55]. In the presence of BMF overexpression, cell apoptosis was induced, whereas conversely, BMF knockdown protected neurons against death by incorporating with other important BH3-only proteins [56]. BMF overexpression causes neuron death and BMF knockdown protects neurons against death evoked by deprivation of nerve growth factor [56]. Moreover, silencing of BMF, in the present study, inhibited apoptosis induced by OGD/RP in vitro and reduced the volume brain lesions, by positively interacting with miR-22-3p.

## Conclusions

**Fig. 8:**
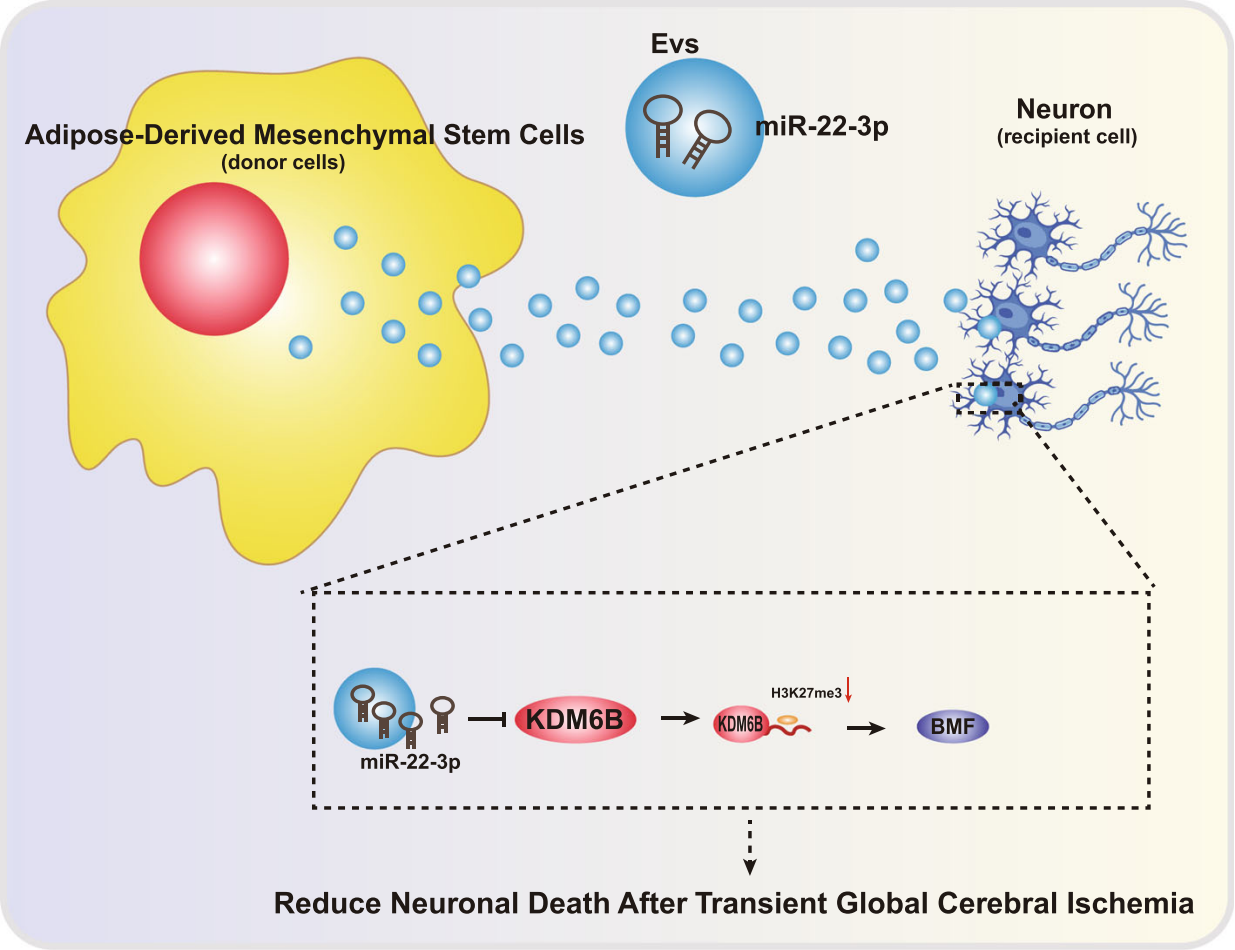
Molecular schematic diagram concerning EV-miR-22-3p in cerebral ischemic injury. ASC-EVs could alleviate cerebral ischemic injury, where miR-22-3p was enriched in ASC-EVs. ASC-EV-miR-22-3p targeted and inhibited KDM6B expression, suppressing the binding between KDM6B and BMP2, and the decreased KDM6B downregulated BMF through the inhibition of BMP2 expression. Thereby, ASC-EV-miR-22-3p attenuates cerebral ischemic injury by suppressing KDM6B-mediated effects on the BMF/BMP2 axis

Our work unraveled an underlying mechanism whereby ASCs could protect cerebral ischemic injury via EV-miR-22-3p by suppressing KDM6B-mediated effects on the BMF/BMP2 axis (Fig. 8). These findings could contribute to the development of a novel strategy of neuroprotection in cerebral ischemic injury.

## Data Availability

The datasets generated/analyzed during the current study are available.

## Abbreviations

MCAO: Middle cerebral artery occlusion
OGD/RP: Oxygen-glucose deprivation/reperfusion
ASC-EVs: ASCs and ASC-derived EVs
MSC: Mesenchymal stem cell
EVs: Extracellular vesicles
